# Author Correction: Microglia-specific overexpression of α-synuclein leads to severe dopaminergic neurodegeneration by phagocytic exhaustion and oxidative toxicity

**DOI:** 10.1038/s41467-021-27737-z

**Published:** 2021-12-16

**Authors:** Simone Bido, Sharon Muggeo, Luca Massimino, Matteo Jacopo Marzi, Serena Gea Giannelli, Elena Melacini, Melania Nannoni, Diana Gambarè, Edoardo Bellini, Gabriele Ordazzo, Greta Rossi, Camilla Maffezzini, Angelo Iannelli, Mirko Luoni, Marco Bacigaluppi, Silvia Gregori, Francesco Nicassio, Vania Broccoli

**Affiliations:** 1 Division of Neuroscience, IRCCS San Raffaele Scientific Institute, 20132 Milan, Italy; 2 Center for Genomic Science of IIT@SEMM, Istituto Italiano di Tecnologia (IIT), 20139 Milan, Italy; 3 National Research Council (CNR), Institute of Neuroscience, 20129 Milan, Italy; 4 Division of Neuroscience, Institute of Experimental Neurology, IRCCS San Raffaele Scientific Institute, Milan, Italy; 5 San Raffaele Telethon Institute for Gene Therapy, IRCCS San Raffaele Scientific Institute, Milan, Italy

**Keywords:** Parkinson's disease, Molecular neuroscience, Microglia

Correction to: *Nature Communications* 10.1038/s41467-021-26519-x, published online 29 October 2021.

The original version of this Article contained an error in the spelling of the author Marco Bacigaluppi, which was incorrectly given as Marco Bagicaluppi. This has now been corrected in both the PDF and HTML versions of the Article.

