# Supplementary material for: Microglia-specific overexpression of α-synuclein leads to severe dopaminergic neurodegeneration by phagocytic exhaustion and oxidative toxicity
Source: Nat Commun. 2021 Oct 29;12:6237. doi: 10.1038/s41467-021-26519-x (PMC8556263; doi:10.1038/s41467-021-26519-x)
Supplement: Supplementary file 1 — Supplementary Information [file 41467_2021_26519_MOESM1_ESM.pdf]

SUPPLEMENTARY INFORMATION

Microglia-specific overexpression of alpha-Synuclein leads to severe dopaminergic neurodegeneration by phagocytic exhaustion and oxidative toxicity

Simone Bido, Sharon Muggeo, Luca Massimino, Matteo Jacopo Marzi, Serena Gea Giannelli, Elena Melacini, Melania Nannoni, Diana Gambarè, Edoardo Bellini, Gabriele Ordazzo, Greta Rossi, Camilla Maffezzini, Angelo Iannelli, Mirko Luoni, Marco Bagicaluppi, Silvia Gregori, Francesco Nicassio, Vania Broccoli.

Supplementary Figure 1

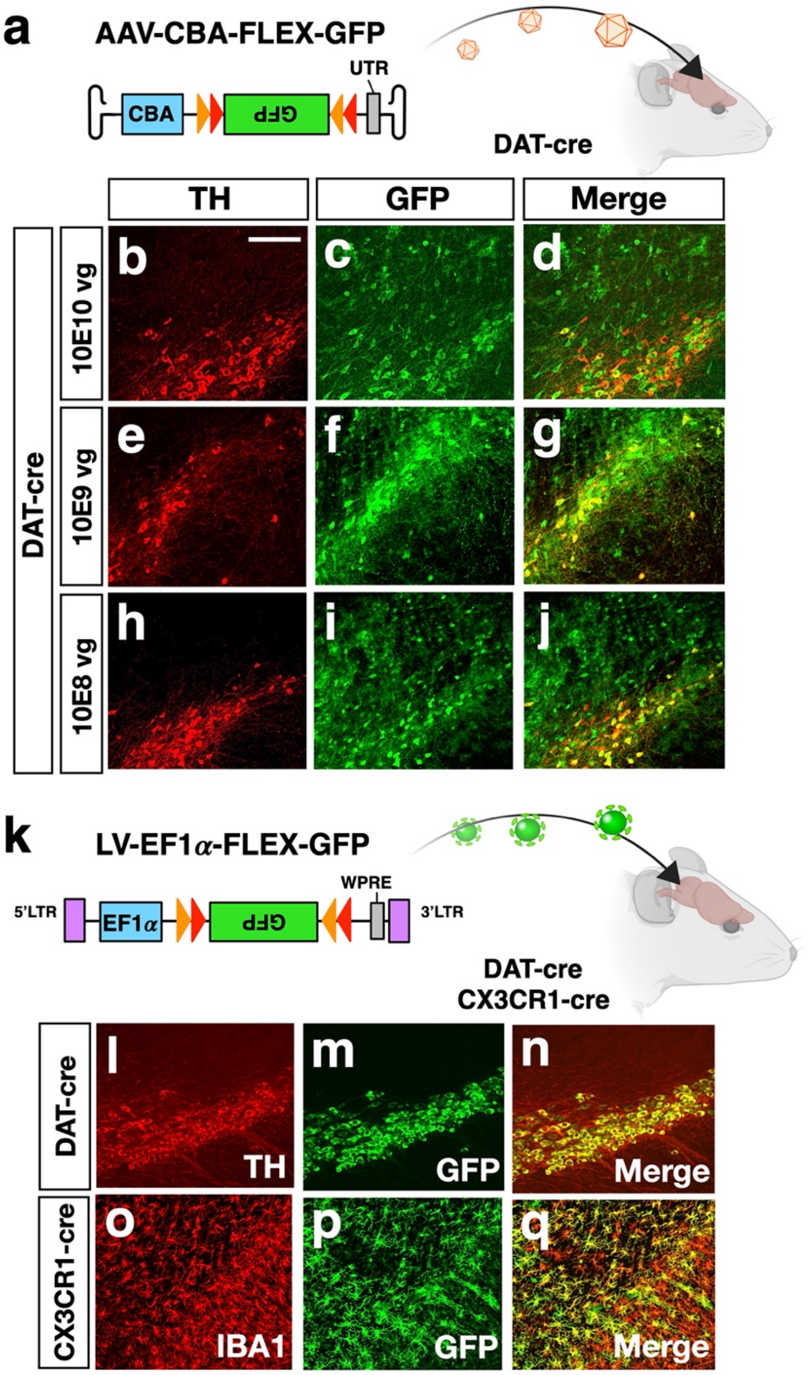

Supplementary Figure 1. Cell specific transgene expression using cre-inducible lentiviral, but not AAV, vectors.

a-j The Cre-inducible FLEX GFP construct packaged in AAV particles produced leaky GFP expression in TH^- cells when injected in DAT-Cre mice at three different dilutions (representative images of n=4 nigrae). **k-q** A similar Cre-inducible vector packaged into lentiviruses showed a strong specificity with GFP expression strictly confined to TH^+ DA neurons and $IBA1^+$ microglial cells when delivered into DAT-Cre and CX3CR1-CreERT2 mice (representative images of n=4 nigrae). Scale bar: 200µm.

Supplementary Figure 2

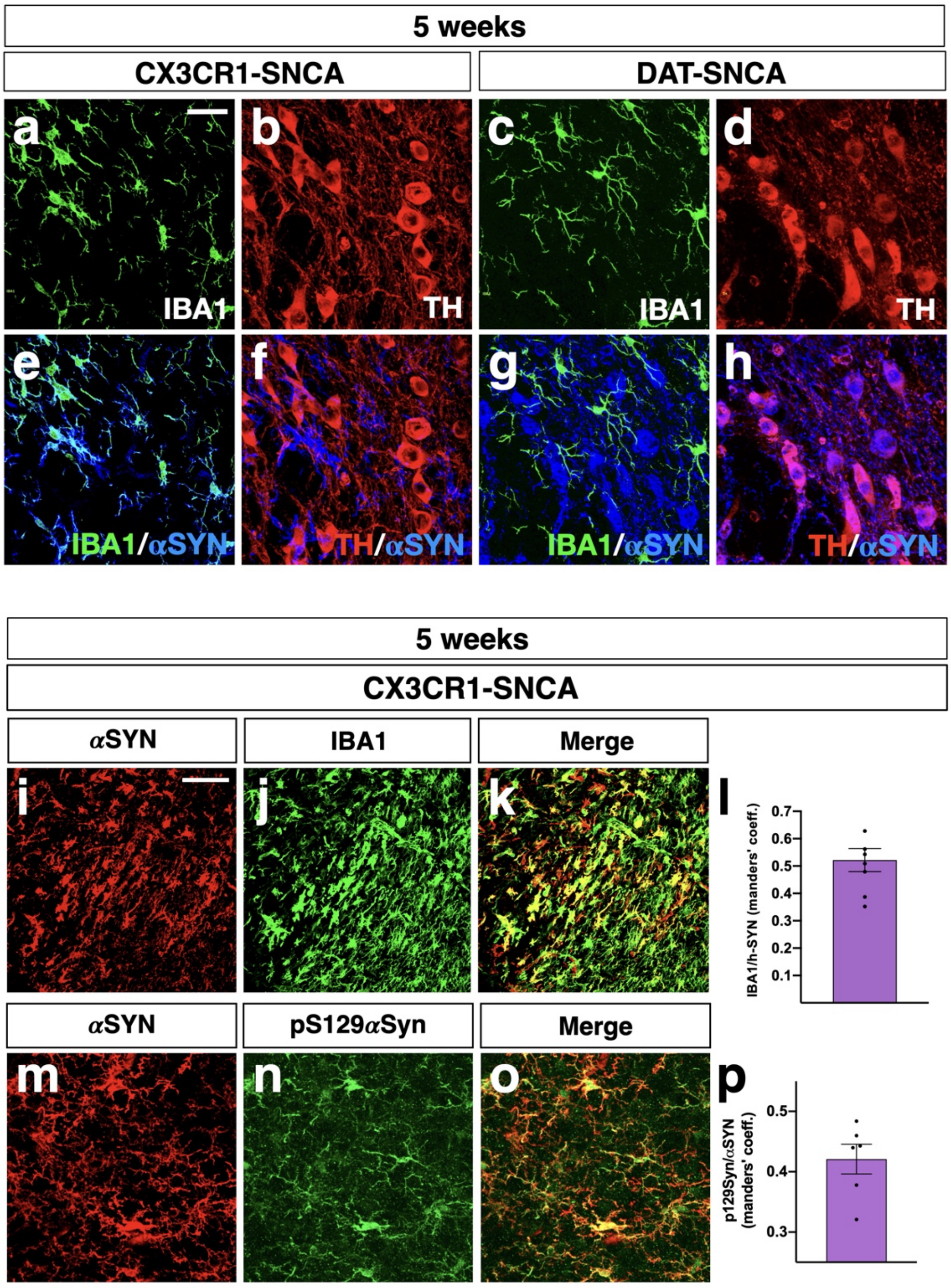

Supplementary Figure 2. Viral αSYN gene expression is limited to targeted cell and leads to αSYN pathological endogenous aggregation in CX3CR1-SNCA mice microglia.
a-h Images showing the exact overexpression of αSYN within the Cre-expressing cells (representative images of n=6 nigrae). **i-l** Representative images of IBA1^{+} cells (green) and viral αSYN gene expression (red) showing a virtual co-expression with 55% ± 3.3% of IBA1^{+} cells expressing the viral transgene. **m-p** Double immunofluorescence for viral αSYN gene expression (red) and pS129-αSYN (green) showing that 42% ± 4.1% of microglia exhibit pathological αSYN aggregates. Data are the mean ± s.e.m from n=6 nigral sample tissues. Scale bar, 45μm.

Supplementary Figure 3

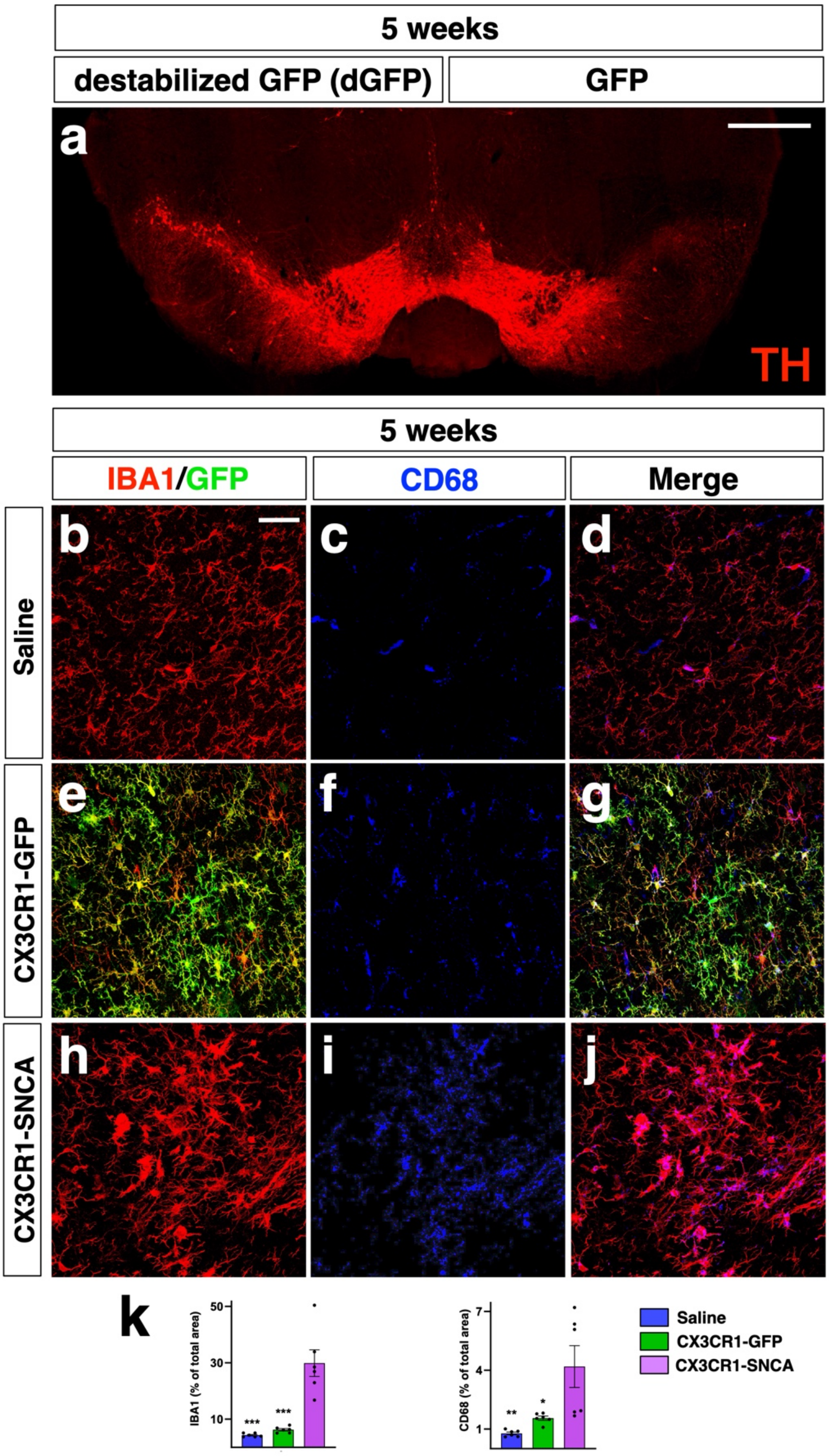

Supplementary Figure 3. The LV mediated GFP overexpression in microglia does not affect its homeostatic state but it provokes neurodegeneration when expressed in DA neurons.

a Representative image of n=3 animals showing the neurodegeneration caused by GFP overexpression in DA neurons compared to the destabilized GFP (dGFP). **b-k** The neuroinflammation in CX3CR1-cre animals (measured as the area occupied by IBA1 and CD68 signal), is not affected by the GFP overexpression compared with saline injected mice. Conversely, the overexpression of αSYN causes the activation of microglia as indicated by the increase of both IBA1 and CD68 staining. Data are the mean ± s.e.m from n = 6 nigral tissue samples. One-way ANOVA is followed by Bonferroni's post-test. Panel **k** (left): ***p<0.0001 (vs. CX3CR1-SNCA). Panel **k** (right): * and ** indicate respectively p=0.026 and p=0.004 vs. CX3CR1-SNCA. Scale bar, 45μm.

Supplementary Figure 4

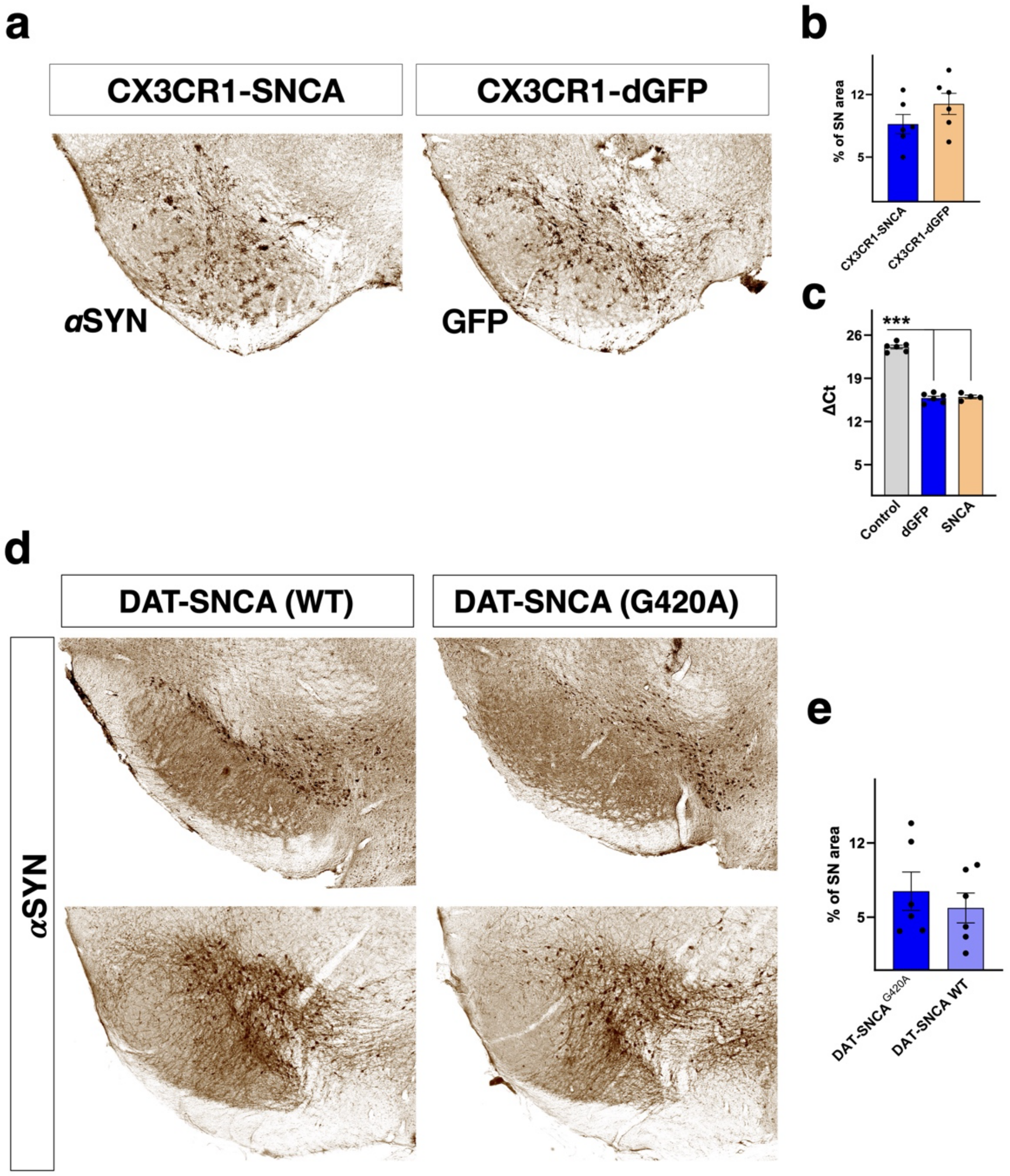

Supplementary Figure 4. Analysis of transgene expression levels in brain tissues.
a-c Protein and transcript levels of the SNCA and dGFP transgenes as revealed respectively by immunohistochemistry (8.6 ± 1.0 and 10.9 ± 1.2 for SNCA and dGFP proteins, respectively and by qPCR (ΔCt of 15.8 ± 0.3 and 16 ± 0.3 for dGFP and SNCA gene, respectively) in either CX3CR1-SNCA or CX3CR1-dGFP animals. Panel **b**: Two tailed unpaired t test (t=1.4, df=10) revealed no differences between groups (p=0.18). Panel **c**: one-way ANOVA is followed by Bonferroni's post-test. ***p<0.0001 (vs. control). **d,e** Levels of the wild type (SNCA-WT) or mutated ($SNCA^{G420A}$) SNCA protein are not significantly different when assessed by immunohistochemistry in nigral tissues of transduced mice (7,4 ± 1.8 and 5.9 ± 1.4 for $SNCA^{G420A}$ and $SNCA^{WT}$, respectively. Two tailed unpaired t test (t=0.68, df=10) revealed no differences between groups (p=0.5). Data are the mean ± s.e.m from n = 6 nigrae. Scale bar, 300 μ

Supplementary Figure 5

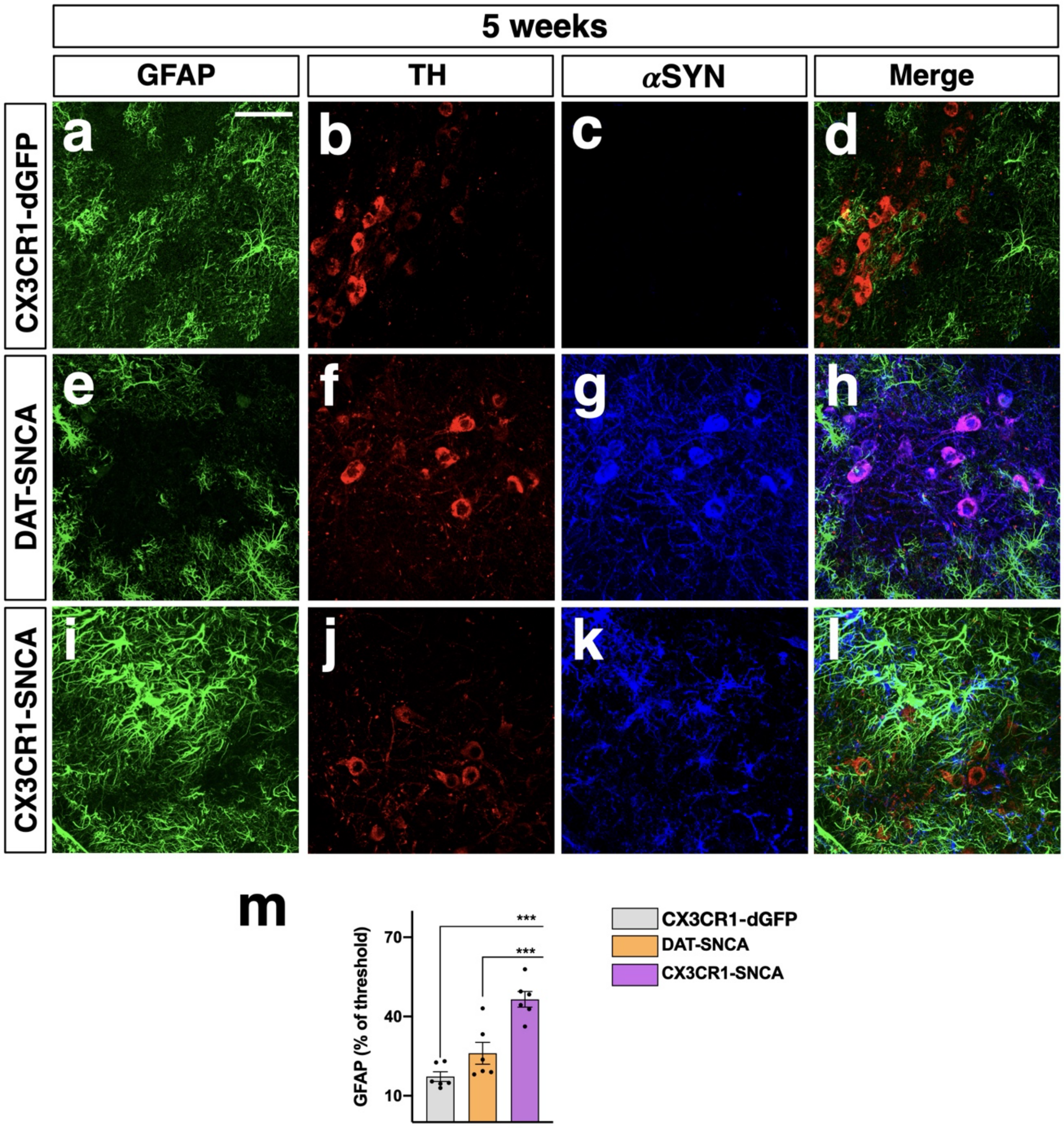

Supplementary Figure 5. Astrocyte activation in CX3CR1-SNCA mice.
a-m Significant increase in GFAP immunofluorescence signal, as measured by the occupied area of a fixed signal threshold, indicates aberrant astrocyte activation in CX3CR1-SNCA, but not in DAT-SNCA, relative to CX3CR1-dGFP mice. Data are the mean ± s.e.m from n = 6 nigral tissue samples. One-way ANOVA is followed by Bonferroni's post-test. *** indicates $p < 0.0001$ (CX3CR1-dGFP vs. CX3CR1-SNCA) and $p = 0.001$ (DAT-SNCA vs. CX3CR1-SNCA). Scale bar, 45μm.

Supplementary Figure 6

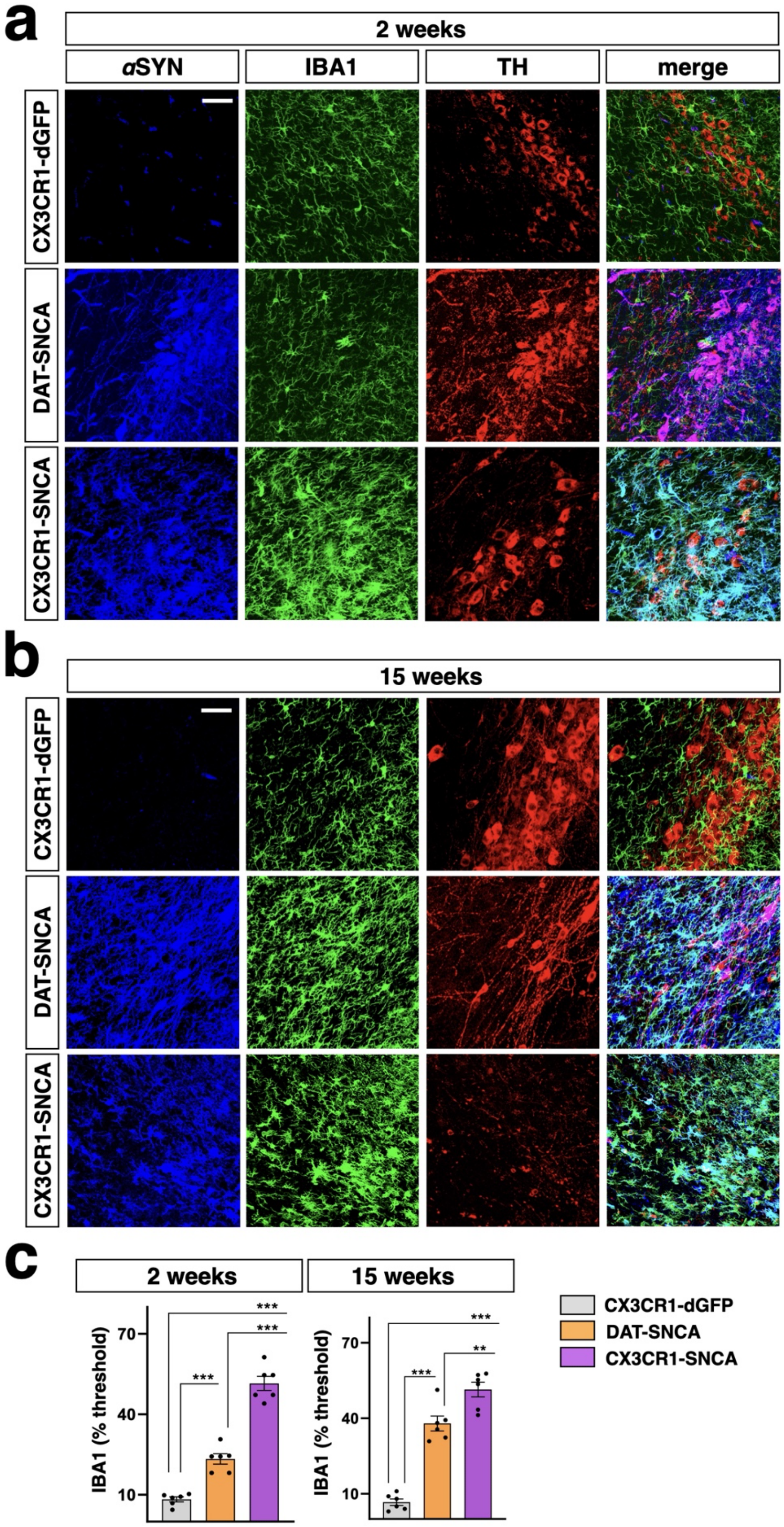

Supplementary Figure 6. Neuroinflammation appears early at 2 weeks and persists after 15 weeks from injection.

a-c IBA1 immunostaining (in green) indicates a significant increase of neuroinflammation (as measured by the occupied area of a fixed signal threshold) in DAT-SNCA and CX3CR1-SNCA mice 2 weeks from injection, with the latter group showing a greater extent of microgliosis. After 15 weeks, DAT-SNCA animals display an increase of IBA1 signal whereas the microgliosis in CX3CR1-SNCA results stable. Data are the mean ± s.e.m from n = 6 nigral tissue samples. One-way ANOVA is followed by Bonferroni's post-test. Left bar graph: *** indicates $p<0.0001$ and $p = 0.0002$ (CX3CR1-dGFP vs. DAT-SNCA). Right bar graph: *** and ** indicate $p<0.0001$ and $p=0.0056$ respectively. Scale bar, 45μm.

Supplementary Figure 7

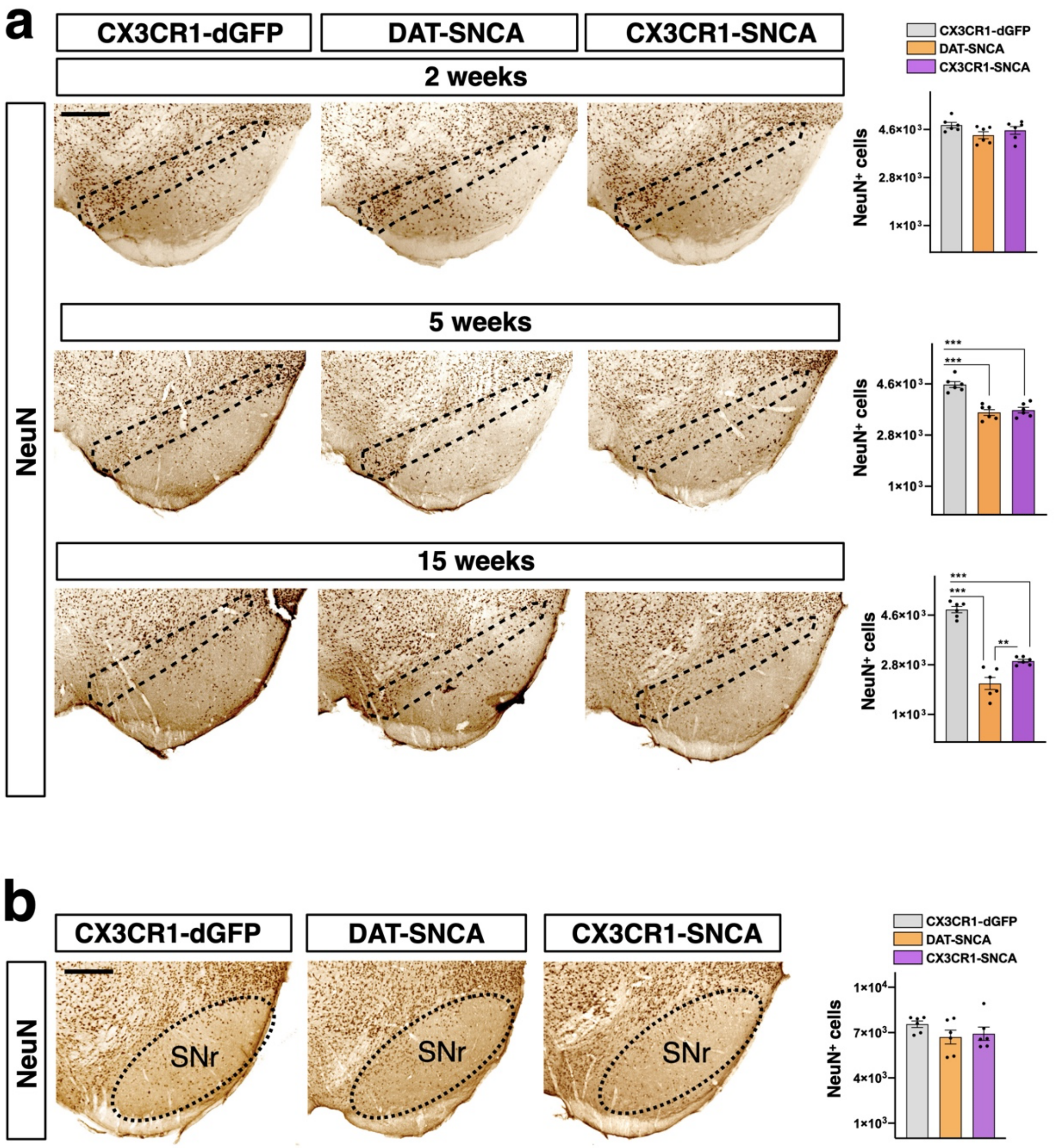

Supplementary Figure 7. αSYN overexpression is leading to the loss of DA neurons without change in the numbers of the *substantia nigra pars reticulata* neurons.

a Unbiased stereological count of the NeuN+ cells reveals neuronal loss in the substantia nigra pars compacta (highlighted area) at 5 and 15 weeks after LV injection. At 5 weeks the cell loss is roughly 21% ± 0.8 and 20% ± 0.8 in DAT-SNCA and CX3CR1-SNCA, respectively. At 15 weeks the neurodegeneration is more severe reaching 54% ± 1.7 and 36% ± 0.5 in DAT-SNCA and CX3CR1-SNCA mice, respectively. **b** Conversely, the number of the NeuN+ cells in the substantia nigra pars reticulata (SNR, highlighted area) is comparable in DAT-SNCA and CX3CR1-SNCA mice at 5 weeks after viral injection. Data are the mean ± s.e.m from n=6 nigral tissue samples. One-way ANOVA is followed by Bonferroni's post-test. Panel **a,** upper bar graph: no significant effect of treatment ($F_{2,15}$=2,230, p=0.1420); middle bar graph: *** p<0.0001; lower bar graph *** and ** indicate p<0.0001 and p=0.004 respectively. Panel **b**: no significant effect of treatment ($F_{2,15}$=1,299, p=0.30).

Supplementary Figure 8

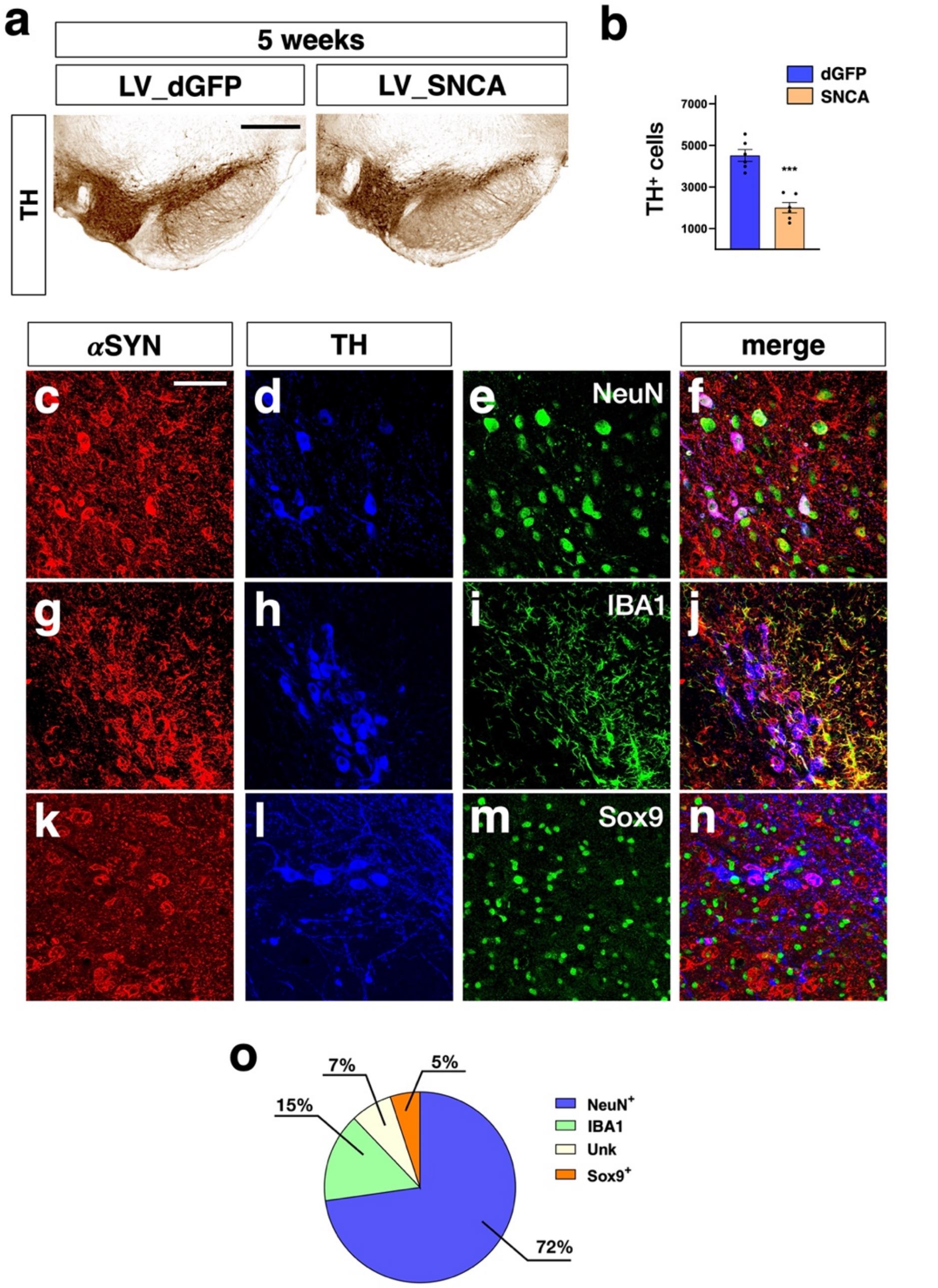

Supplementary Figure 8. Simultaneous αSYN overexpression in neurons and glia exacerbates the neurodegeneration in the nigral tissue.

a,b The simultaneous overexpression of αSYN in neuronal and glial cells achieved by injecting wild type mice with a lentivirus expressing SNCA under a constitutive promoter leads to a severe neurodegeneration. At 5 weeks the LV_SNCA injected mice show 55% ± 2% of neurodegeneration *in the substantia nigra* (SN) compared to LV_dGFP injected animals. Data are the mean ± s.e.m from n = 6. ***p<0.0001 calculated with the unpaired two-tailed Student T test (t=6.624, df=10). Scale bar, 45 µm. **c-n** Representative co-stainings between aSYN and selective markers of cellular sub-types in the brain (n = 6 independent nigrae). **o** Percentage of neurons (NeuN+), microglia (IBA1+) and astrocytes (Sox9+) that express the human αSYN. Scale bar, 300µm.

Supplementary Figure 9

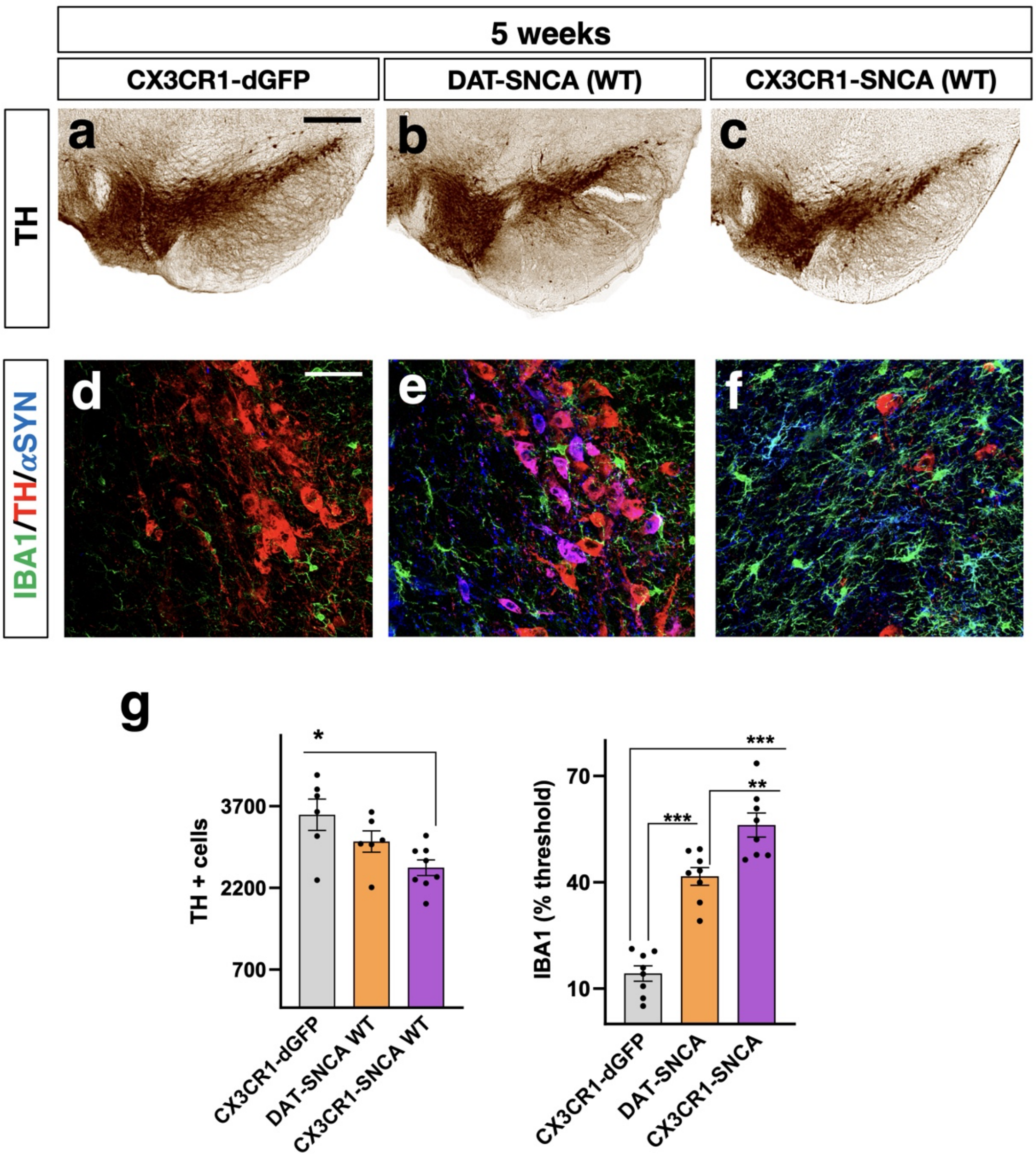

Supplementary Figure 9. Wild type αSYN overexpression causes significant neuroinflammation accompanied by DA neuronal loss in CX3CR1-cre animals. TH immunostaining showing the significant neurodegeneration caused by wild type αSYN expression in microglia (CX3CR1-SNCA-WT), but not in SA neurons (DAT-SNCA-WT) at 5 weeks after viral transduction. **a-g** The unbiased stereological counts confirm a significant loss of TH^+ neurons in the nigral tissue (24% ± 1.8 %) in CX3CR1-SNCA-WT animals and a mild cell loss in DAT-SNCA-WT mice (11% ± 2%). The neurodegeneration is accompanied by a significant increase of neuroinflammation measured as IBA1 (green) signal in CX3CR1-SNCA-WT. Data are the mean ± s.e.m from n=6 nigral tissue samples. One-way ANOVA is followed by Bonferroni's post-test. Left bar graph: *p=0.01; right bar graph: * and ** indicate p=0.016 and p=0.0012, respectively. Scale bar, 300μm (**a,b,c**); 45μm (**d,e,f**).

Supplementary Figure 10

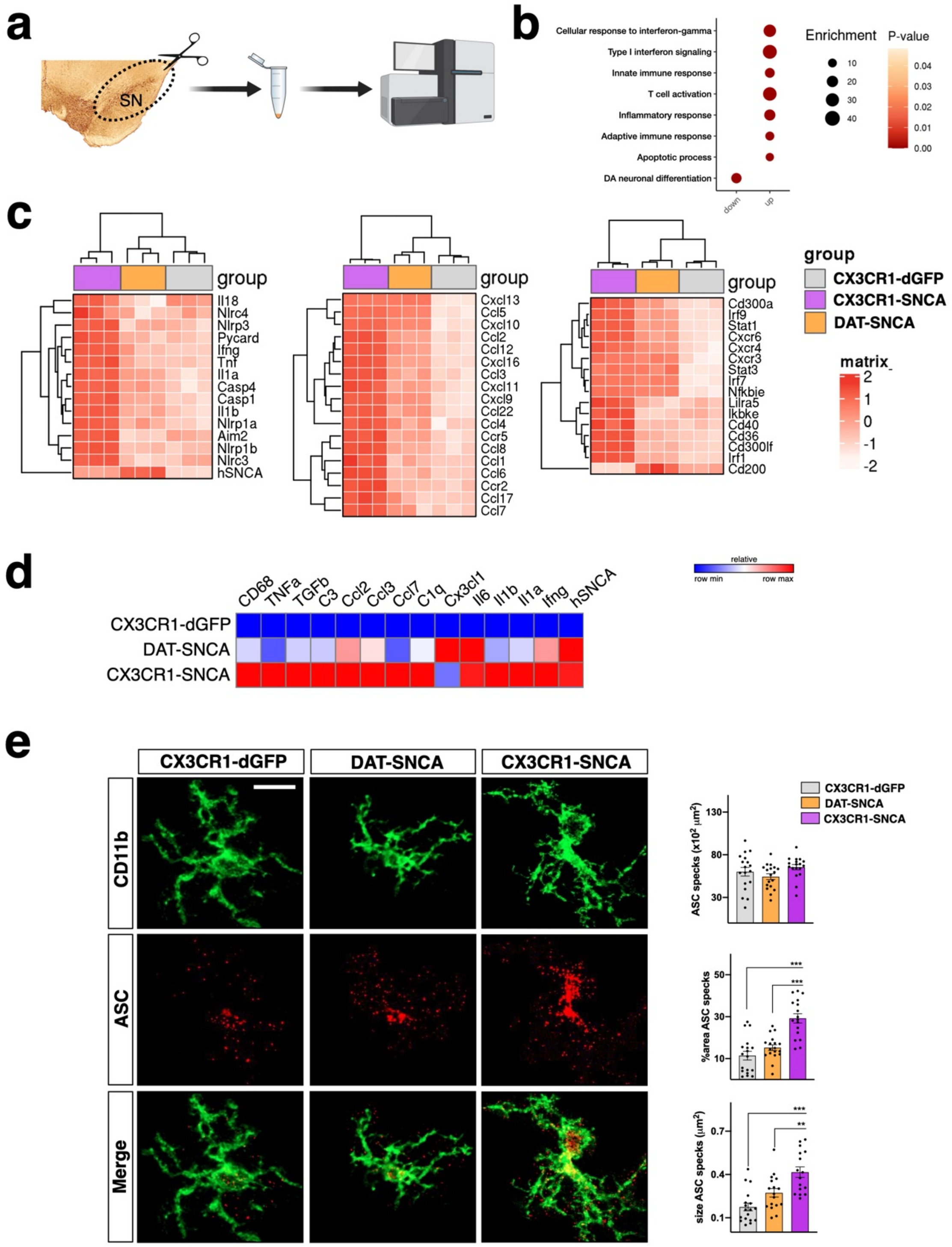

Supplementary Figure 10. Bulk RNA-seq analysis and expression of cytokines, ROS and inflammasome related genes in CX3CR1-SNCA mice.
a Fresh dissected *substantia nigrae* (SN) from CX3CR1-dGFP, DAT-SNCA and CX3CR1-SNCA animals were isolated for RNA extraction and subsequent bulk RNA sequencing. **b** Top canonical pathways identified by IPA that are differentially expressed between CX3CR1-SNCA mice compared to the other two groups. **c** Heatmaps showing the different level of expression of the selected genes in the 3 experimental groups. **d** Real-time PCRs for independent validation

of the differently expression of the principal cytokines and inflammation markers among the three mouse groups. **e** ASC specks localization, size, and abundance in CD11b positive cells in CX3CR1-dGFP, DAT-SNCA and CX3CR1-SNCA animals. The corresponding quantification shows a significant increase of the area occupied by ASC only in CX3CR1-SNCA as a consequence of the increase in size but not in the number of specks. The data are collected 5 weeks after viral injection. Data are the mean ± s.e.m from n=18 cells from 6 different SN. One-way ANOVA is followed by Bonferroni's post-test. Panel **e**, upper bar graph: no significant effect of treatment ($F_{2, 51}$=1,993, p=0.14); middle bar graph *** p<0.0001; lower bar graph: ** and *** indicate p= 0.0062 and p<0.0001, respectively. Scale bar, 12μm.

Supplementary Figure 11

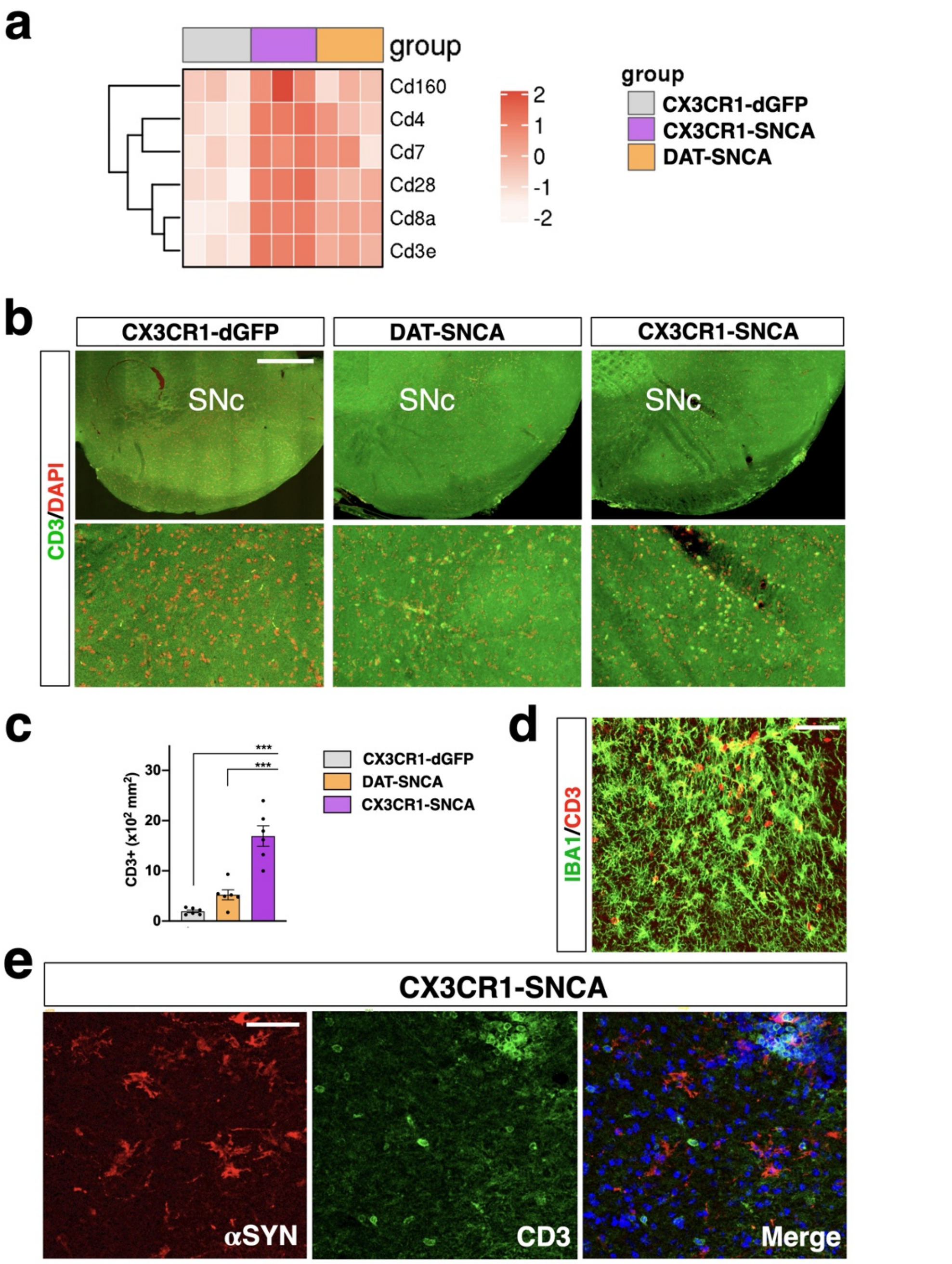

Supplementary Figure 11. Brain recruitment of T cells in DAT-SNCA and CX3CR1-SNCA mice.

a Heatmap showing the expression levels deducted by the bulk RNA-seq datasets of $CD4^+$ and $CD8^+$ T cell specific genes. **b,c** Immunofluorescence for CD3 highlighting the particular abundance of T lymphocytes (green) infiltrated in the brain nigral tissue (SN) in CX3CR1-SNCA mice compared with the other experimental groups. Data are the mean ± s.e.m from n=6 nigral samples. One-way ANOVA is followed by Bonferroni's post-test. ***$p<0.0001$. Scale bar, 200 µm. **d** High power view (representative of n=6 independent nigrae) showing the close proximity of $IBA1^+$ microglia and other myeloid cells (green) with infiltrated T lymphocytes (red). Scale bar, 45 µm. **e** Double immunofluorescence for αSYN and CD3 (representative images of n=6 independent nigrae) showing that infiltrated T cells in the nigral tissue do not exhibit expression or uptake of the AAV expressed human αSYN protein. Scale bar, 100µm.

Supplementary Figure 12

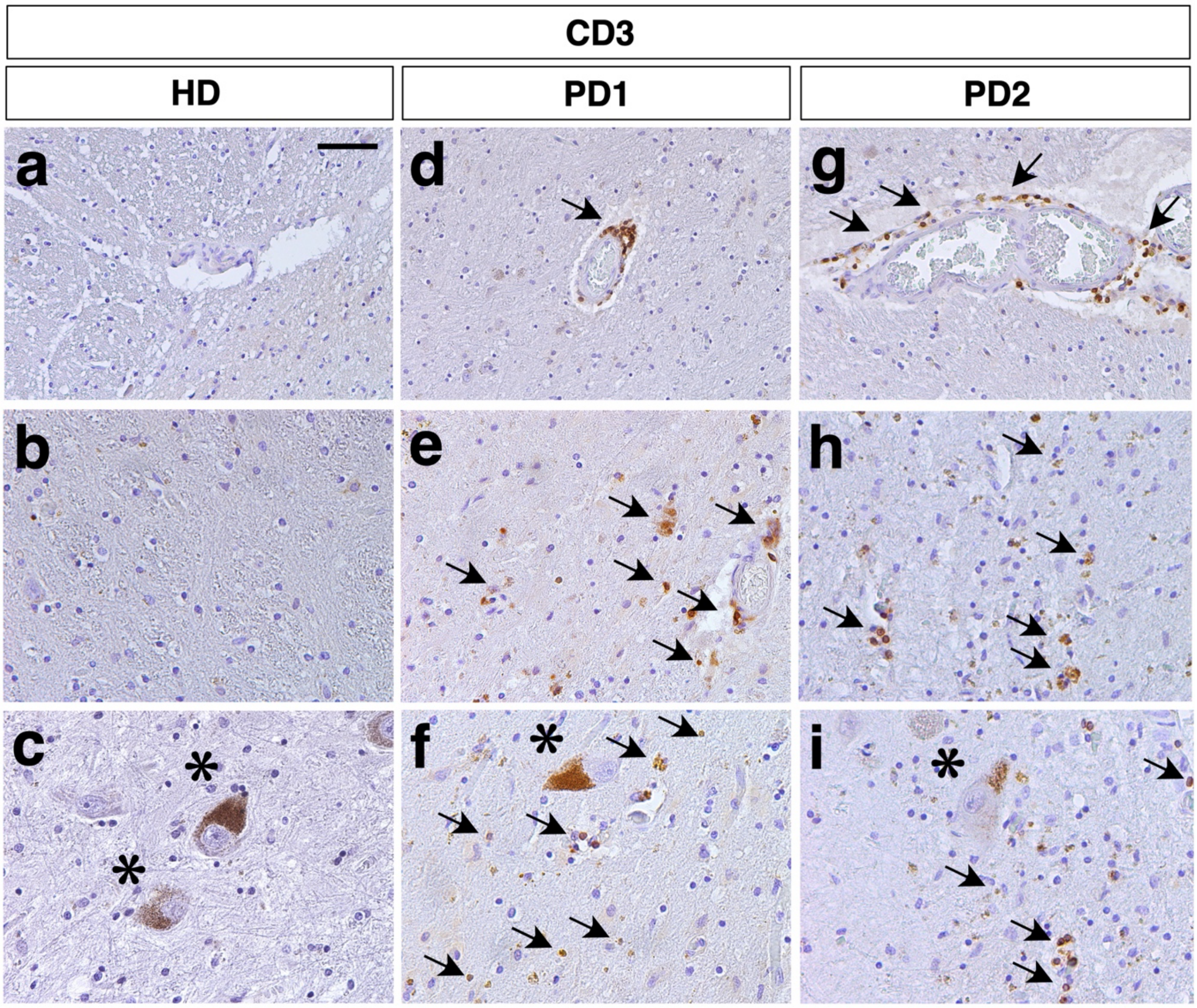

Supplementary Figure 12. CD3 immunohistochemistry on autoptic human brain nigral tissues.

a-i CD3 immunostaining reveal the presence of numerous perivascular and parenchymal T lymphocytes (black arrows) in close proximity to neuromelanin positive neurons (black stars) in PD patient (PD) (**d-i**), but not healthy donor (HD)(**a-c**), brain nigral samples (representative images of n=4 independent brain samples from healthy donor and n=12 independent PD brain samples). Scale bar, 25µm.

Supplementary Figure 13

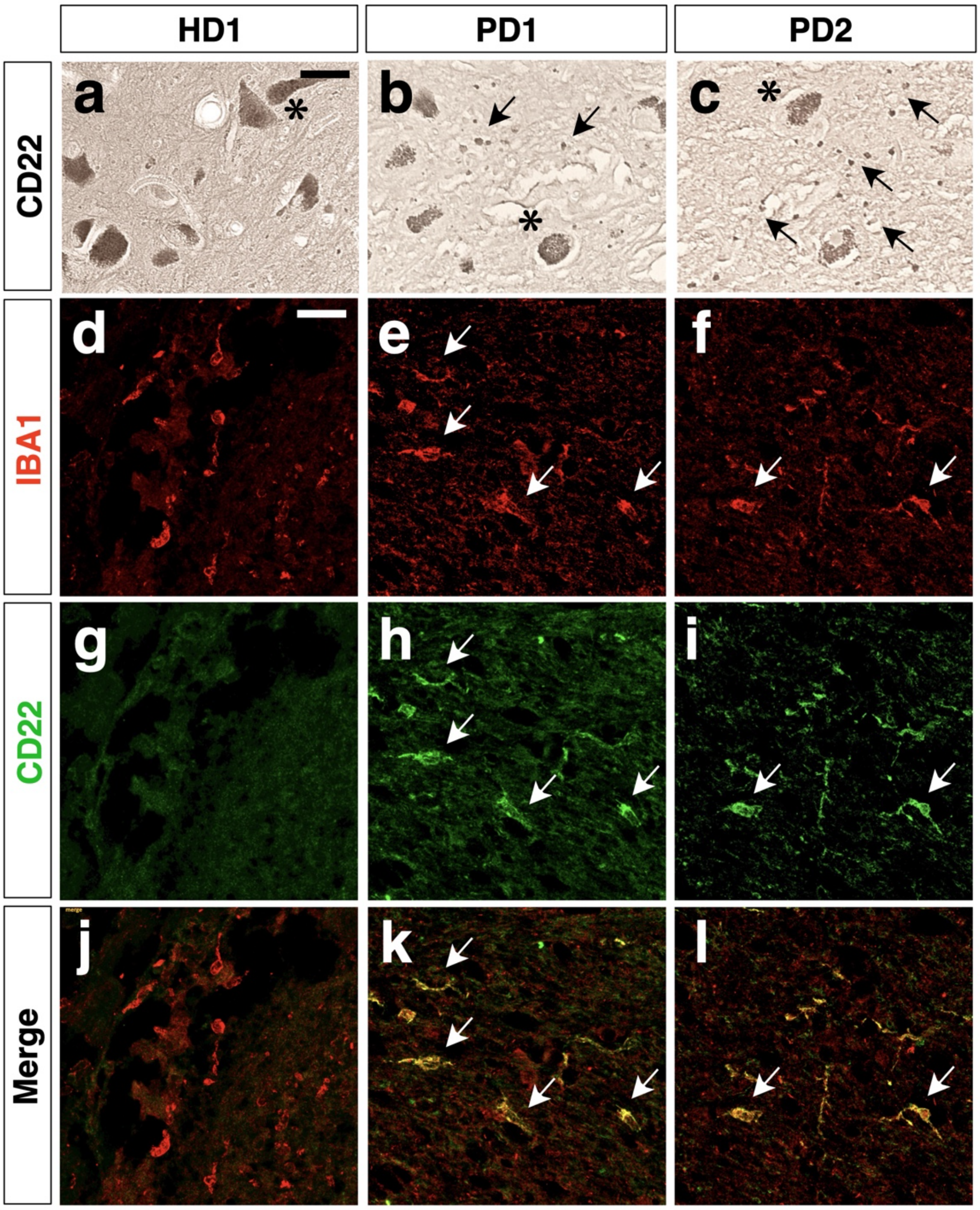

Supplementary Figure 13. CD22 immunohistochemistry on autoptic human brain nigral tissues.

a-c Immunohistochemistry on human brain nigral tissue from healthy control (HD) (**a**) and PD-diagnosed patients (PD) (**b,c**) detected the presence of CD22^{+} cells (black arrows) in close proximity with neuromelanin positive neurons (black stars) in pathological samples. **d-l** Double immunofluorescence confirms the colocalization of CD22 (red) with IBA1^{+} cells (red) (representative images of n=4 independent brain samples from healthy donor and n=12 independent PD brain samples). Scale bar, 25μm.

Supplementary Figure 14

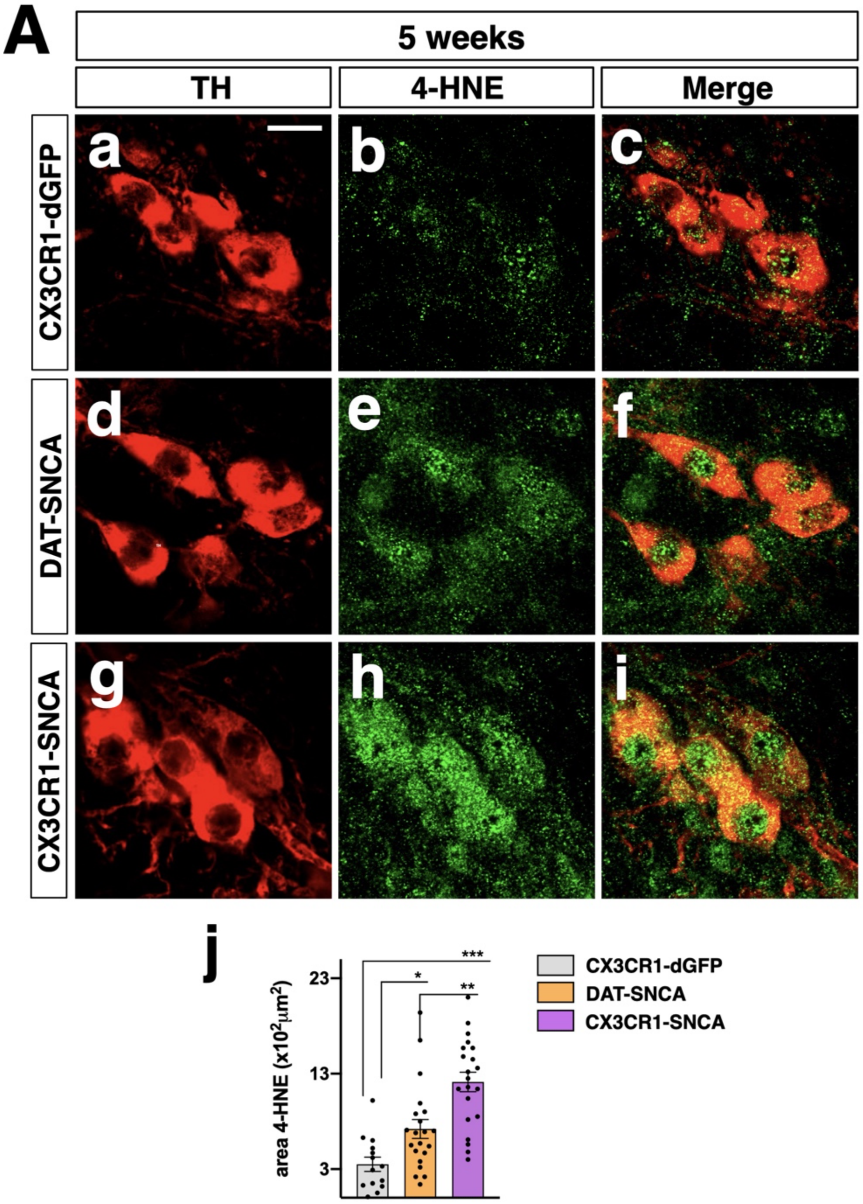

Supplementary Figure 14. 4-HNE levels in DA neurons of DAT-SNCA and CX3CR1-SNCA mice.

a-i Double immunofluorescence for the 4-HNE oxidative marker (green) and TH (red) reveals heightened 4-HNE levels in the nigral DA neurons of DAT-SNCA and CX3CR1-SNCA mice. (**j**) Quantifications were carried out by measuring the area occupied by a given 4-HNE signal threshold in TH+ domains. Data are the mean ± s.e.m from n = 14-21 cells collected from 6 nigral samples. One-way ANOVA is followed by Bonferroni's post-test. *, ** and ***, indicate p=0.043, p=0,0012 and p<0.0001, respectively. Scale bar, 15μm.

Antibody	Cat. Number	Host	Dilution	Company
4-HNE	HNE11-S	Rabbit	1:100	alpha diagnostic
ASC	AL177	Rabbit	1:250	Adipogen
CD11b efluor 450	48-0112-82	Rat	1:200	eBioscience
CD22	EPR20061	Rabbit	1:250	Abcam
CD3	MCA772GA	Rat	1:100	Bio-rad
CD45 PerCP-Cyanine 5.5	45-0451-82	Rat	1:200	eBioscience
CD68	MCA1957T	Rat	1:250	Bio-rad
GFAP	GA524	Rabbit	1:1000	Dako
GFP	AB13970	Chicken	1:1000	Abcam
GFP	AB290	Rabbit	1:1000	Abcam
human alpha-synuclein	180215	Mouse	1:100	Thermo Fisher
IBA1	019-19741	Rabbit	1:1000	Wako
IBA1	234006	Chicken	1:1000	Synaptic System
NeuN	EPR12763	Rabbit	1:500	Abcam
p62/SQSTM1	NBP1-48320	Rabbit	1:500	Novus
pS129 alpha-synuclein	EP1536Y	Rabbit	1:1000	Abcam
Tyrosine Hydroxylase	AB76442	Chicken	1:500	Abcam
Tyrosine Hydroxylase	AB112	Rabbit	1:500	Abcam

Supplementary Table 1: List of the primary antibodies used in this study.

**Il1beta**	Fw	AGCTCTCCACCTCAATGGAC
	Rev	GCCGTCTTTCATTACACAGG
**Ccl2**	Fw	GGCTGGAGAGCTACAAGAGG
	Rev	ATGTCTGGACCCATTCCTTC
**Tnfα**	Fw	GGGCTTCCAGAACTCCAG
	Rev	GGGCCATAGAACTGATGAGAG
**Il6**	Fw	TGGATGCTACCAAACTGGAT
	Rev	TCTGAAGGACTCTGGCTTTG
**Tgfβ**	Fw	GGAATACAGGGCTTTCGATT
	Rev	CCATGAGGAGCAGGAAGG
**Cx3cl1**	Fw	ATGACCTCACGAATCCCAGT
	Rev	CTTGGACCCATTTCTCCTTC
**Il10**	Fw	GGCCCAGAAATCAAGGAG
	Rev	TCACTCTTCACCTGCTCCAC
**Cd68**	Fw	CAGGGAGGTTGTGACGGTA
	Rev	CGCCATGTAGTCCAGGTAGA
**Grn**	Fw	GACCTGGTTCACACACGATG
	Rev	AGAATCATCGGGACACTGGG
**Ardb1**	Fw	CATCGTTCTGCTCATCGTGG
	Rev	GGCCAGGGACATGATGAAGA
**Ardb2**	Fw	TGCCACCCACAAGAAAGCTA
	Rev	GGAAGACCCGGGAATAGACA
**Vps35**	Fw	GCTGCTGGAGAAATTGGCTT
	Rev	GCACACTGAGTTCTCAAGGG
**Slc33a1**	Fw	ACTAGCTGTCCCAATGGTCC
	Rev	TCCACCAAACAAGTAGAGCA
**Mrc1**	Fw	AACAAAGGGACGTTTCGGTG
	Rev	TCCTTCTGCCCAATGTTTGC
**Cyba**	Fw	TTGCCAGTGTGATCTATCTGC
	Rev	GTTGGTAGGTGGTTGCTTGA
**Cybb**	Fw	GTGAGAGGTTGGTTCGGTTT
	Rev	CCTCCATCTTGAATCCCTTC
**Ptgs2**	Fw	CAGCACTTCACCCATCAGTT
	Rev	GGCGCAGTTTATGTTGTCTG
**Nos1**	Fw	AAGGAGATCGAGAGCACCAG
	Rev	GCGTACTTGACGTGGTTACAG
**Nos2**	Fw	TGTGTTCCACCAGGAGATGT
	Rev	CATGACCTTTCGCATTAGCA
**Cd22**	Fw	CCAGGAGAGTCGGAAGAAGT
	Rev	GTTTGTTGCACTTGGACTGG
**Arg1**	Fw	AATGAAGAGCTGGCTGGTGT
	Rev	GACACATAGGTCAGGGTGGA
**18S**	Fw	GTAACCCGTTGAACCCCATT
	Rev	CCATCCAATCGGTAGTAGCG

Supplementary Table 2. Sequence of the primers for qPCRs.

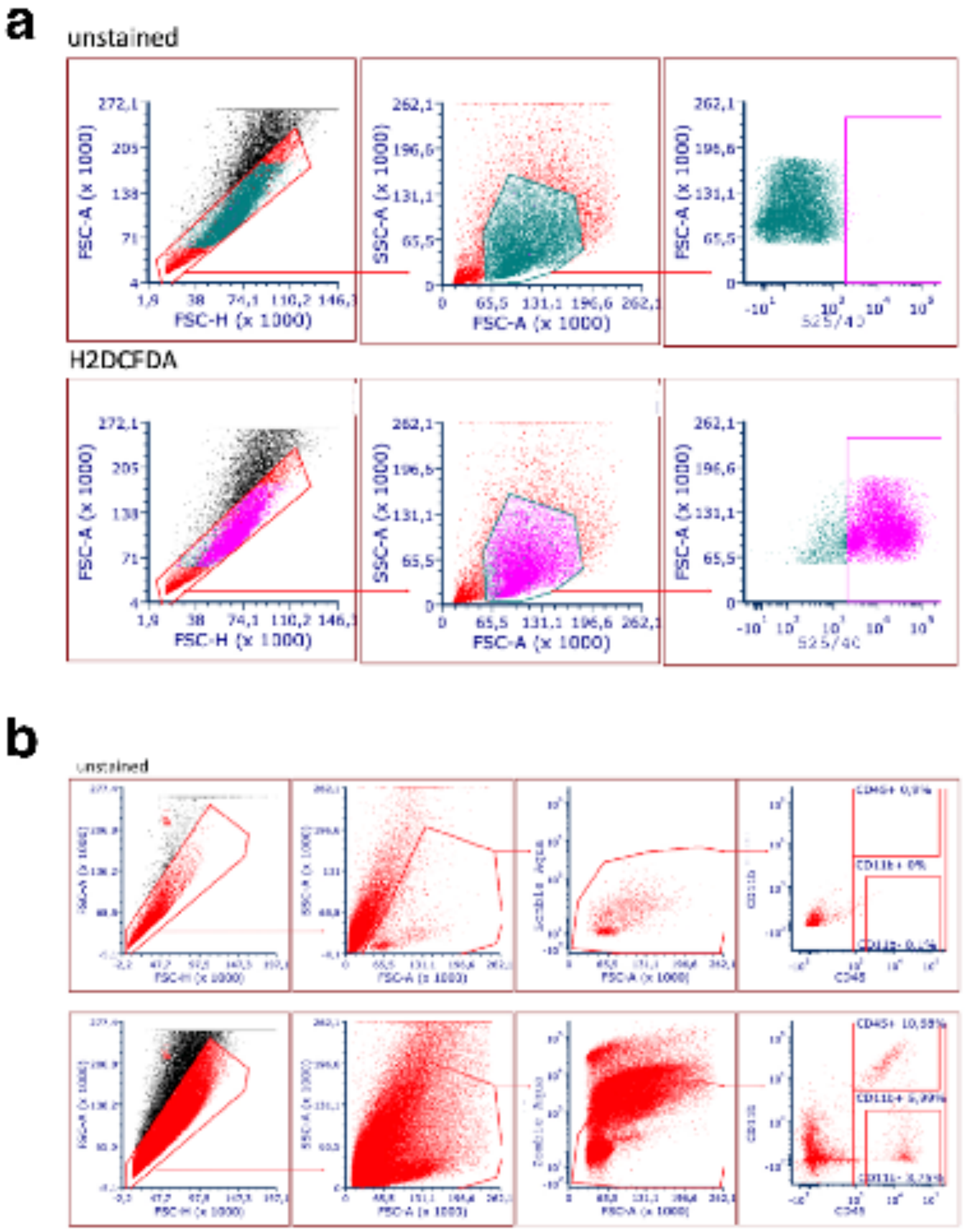

Supplementary Table 3: a, Gating strategy related to Fig.6g. H2DCFDA dot plots were gated on scatters population after doublets exclusion. Unstained samples were employed to set gates. **b**, Gating strategy related to Fig.4a. CD45+ cells, divided in CD11b+ and CD11b-, were identified employing unstained samples to set gates. Dead cell discrimination was performed on scatters population after doublets exclusion.
