# Supplementary material for: Microglia-specific overexpression of α-synuclein leads to severe dopaminergic neurodegeneration by phagocytic exhaustion and oxidative toxicity
Source: Nat Commun. 2021 Oct 29;12:6237. doi: 10.1038/s41467-021-26519-x (PMC8556263; doi:10.1038/s41467-021-26519-x)
Supplement: Supplementary file 4 — Reporting Summary [file 41467_2021_26519_MOESM4_ESM.pdf]

nature research

Corresponding author(s): Vania Broccoli

Last updated by author(s): Sep 19, 2021

Reporting Summary

Nature Research wishes to improve the reproducibility of the work that we publish. This form provides structure for consistency and transparency in reporting. For further information on Nature Research policies, see our Editorial Policies and the Editorial Policy Checklist.

Statistics

For all statistical analyses, confirm that the following items are present in the figure legend, table legend, main text, or Methods section.

n/a	Confirmed	
[ ]	[x]	The exact sample size (*n*) for each experimental group/condition, given as a discrete number and unit of measurement
[ ]	[x]	A statement on whether measurements were taken from distinct samples or whether the same sample was measured repeatedly
[ ]	[x]	The statistical test(s) used AND whether they are one- or two-sided<br>*Only common tests should be described solely by name; describe more complex techniques in the Methods section.*
[x]	[ ]	A description of all covariates tested
[x]	[ ]	A description of any assumptions or corrections, such as tests of normality and adjustment for multiple comparisons
[ ]	[x]	A full description of the statistical parameters including central tendency (e.g. means) or other basic estimates (e.g. regression coefficient) AND variation (e.g. standard deviation) or associated estimates of uncertainty (e.g. confidence intervals)
[ ]	[x]	For null hypothesis testing, the test statistic (e.g. *F*, *t*, *r*) with confidence intervals, effect sizes, degrees of freedom and *P* value noted<br>*Give P values as exact values whenever suitable.*
[x]	[ ]	For Bayesian analysis, information on the choice of priors and Markov chain Monte Carlo settings
[x]	[ ]	For hierarchical and complex designs, identification of the appropriate level for tests and full reporting of outcomes
[x]	[ ]	Estimates of effect sizes (e.g. Cohen's *d*, Pearson's *r*), indicating how they were calculated

Our web collection on statistics for biologists contains articles on many of the points above.

Software and code

Policy information about availability of computer code

Data collection	Images were acquired with Leica TCS SP8 confocal microscope, MAVIG RS-G4 confocal microscope and with Nikon Eclipse Ni.<br>All the images were analyzed by using ImageJ 1.53 (NIH, USA). In particular the colocalization measurement was carried out with JACop plugin (BIC, Bordeaux, France). Fractal analysis was performed by using FracLac 2.5 (Charles Sturt University, Bathurst, Australia).<br>The cell counting was performed by using Leica DM400B motorized microscope equipped with Stereo Investigator software v. 2018 (MBF Bioscience).<br>Single cell dissociation was performed using Adult Brain Dissociation Kit and OctoMACS Separator (Miltenyi Biotec)<br>Cells were sorted by means of FACSAria Fusion cell sorter equipped with FACSDiva software v 8.0<br>Single cell dissociation was performed using Adult Brain Dissociation Kit and OctoMACS Separator (Miltenyi Biotec)<br>Single cell sequencing through the Chromium platform (10X Genomics) and Libraries were sequenced on a Novaseq 6000 flowcell (Illumina). We used Seurat 4 for downstream computational analyses.<br>With regards to bulk-RNA sequencing the libraries were sequenced on Illumina HiSeq 3000. The genome was aligned with STAR v. 2.7.9<br>For the nitric oxide assay the fluorescence was detected by VICTOR3 Multilabel plate reader (PerkinElmer).<br>Data regarding the DCF assay were collected by using FACSCANTOII (BD Bioscience) and analyzed with FCS Express 7 (De Novo software)
Data analysis	All the statistical analysis was carried out with Prism 8 software (GraphPad software) using one-way/two-way ANOVA followed by Bonferroni's post-test. For experimental designs with less than three groups and one variable we used the unpaired Student T test. In case of non-normal distribution, we applied the Kurskall Wallis test followed by two stage step-up method.

For manuscripts utilizing custom algorithms or software that are central to the research but not yet described in published literature, software must be made available to editors and reviewers. We strongly encourage code deposition in a community repository (e.g. GitHub). See the Nature Research guidelines for submitting code & software for further information.

Data

Policy information about availability of data

All manuscripts must include a data availability statement. This statement should provide the following information, where applicable:

- Accession codes, unique identifiers, or web links for publicly available datasets
- A list of figures that have associated raw data
- A description of any restrictions on data availability

Genomic data are available GEO accession GSE157534, (go to https://www.ncbi.nlm.nih.gov/geo/query/acc.cgi?acc=GSE157534) and GSE157536 (go to https://www.ncbi.nlm.nih.gov/geo/query/acc.cgi?acc=GSE157536)
The corresponding raw data to the figures 2, 3, 5, 6, 7, 8,S2, S3, S4, S5, S6, S7, S8, S9, S10, S11, S14 are publicly available

Field-specific reporting

Please select the one below that is the best fit for your research. If you are not sure, read the appropriate sections before making your selection.

☒ Life sciences ☐ Behavioural & social sciences ☐ Ecological, evolutionary & environmental sciences

For a reference copy of the document with all sections, see nature.com/documents/nr-reporting-summary-flat.pdf

Life sciences study design

All studies must disclose on these points even when the disclosure is negative.

Sample size	With regard to in vivo experiment no statistical methods were used to pre-determine sample sizes, but our sample sizes are similar to those reported in previous publications on mouse models of Parkinson's disease based on viral vector-mediated alpha-synuclein expression (see Deniz K et al., The Journal of Neuroscience, April 1, 2002, 22(7):2780–2791 "Parkinson-Like Neurodegeneration Induced by Targeted Overexpression of alpha-Synuclein in the Nigrostriatal System"; Theodore S et al., Journal of Neuropathology & Experimental Neurology, December 2008, 1149–1158, "Targeted Overexpression of Human α-Synuclein Triggers Microglial Activation and an Adaptive Immune Response in a Mouse Model of Parkinson Disease"; Oliveras-Salva M et al., Molecular Neurodegeneration, 2013 Nov 25;8:44, "rAAV2/7 vector-mediated overexpression of alpha-synuclein in mouse substantia nigra induces protein aggregation and progressive dose-dependent neurodegeneration"; Bourdenx et al., Acta Neuropathologica Communications, 46 (2015), "Lack of additive role of ageing in nigrostriatal neurodegeneration triggered by α-synuclein overexpression".<br>For the in vitro experiments, the sample size was chosen in order to have 80% power of detecting a 33% reduction in the recurrence rate. We assumed that the recurrence rate is normally 30% and hypothesized that our variable (expression of a transgene or treatment) reduces the recurrence rate by the 33% with a significant level of 0.05.
Data exclusions	In this work we included all the data generated from the experiments.
Replication	We repeated all experiments using at least three biological replicates over distinct independent experiments. We specified the number of biological replicates and independent experiments in the respective figure legends.
Randomization	The age and sex matched animals enrolled in the experiments were randomly enrolled in the different experimental groups
Blinding	The investigators who performed the stereological counting and the image acquisitions were blinded to group allocation due to the risk of an unbiased quantification or to an unbiased field acquisition. For all the other experiments the blinding to group allocation was randomly applied and not considered necessary due to the totally automated quantification procedure.

Reporting for specific materials, systems and methods

We require information from authors about some types of materials, experimental systems and methods used in many studies. Here, indicate whether each material, system or method listed is relevant to your study. If you are not sure if a list item applies to your research, read the appropriate section before selecting a response.

Materials & experimental systems

n/a	Involved in the study	
☐	☒	Antibodies
☐	☒	Eukaryotic cell lines
☒	☐	Palaeontology and archaeology
☐	☒	Animals and other organisms
☐	☒	Human research participants
☒	☐	Clinical data
☒	☐	Dual use research of concern

Methods

n/a	Involved in the study	
☒	☐	ChIP-seq
☐	☒	Flow cytometry
☒	☐	MRI-based neuroimaging

Antibodies

Antibodies used	4-HNE HNE11-S Rabbit 1:100 (alpha diagnostics)<br>ASC AL177 Rabbit 1:250 (Adipogen)<br>CD11b eFluor 450 M1/70 Rat 1:200 (eBioscience)<br>CD22 EPR20061 Rabbit 1:250 (Abcam)<br>CD3 MCA772GA Rat 1:100 (Bio-rad)<br>CD45 PerCP-Cyanine 5.5 30-F11 Rat 1: 200 (eBioscience)<br>CD68 MCA1957T Rat 1:250 (Bio-rad)<br>GFAP GA524 Rabbit 1:1000 (Dako)<br>GFP AB13970 Chicken 1:1000 (Abcam)<br>GFP AB290 Rabbit 1:1000 (Abcam)<br>Human alpha-Synuclein (clone LB509) 180215 Mouse 1:100 (Thermo Fisher)<br>Iba1 019-19741 Rabbit 1:1000 (Wako)<br>Iba1 234006 Chicken 1:1000 (Synaptic System)<br>NeuN EPR12763 Rabbit 1:500 (Abcam)<br>p62/SQSTM1 NBP1-48320 Rabbit 1:500 (Novus)<br>pS129 alpha-synuclein EP1536Y Rabbit 1:1000 (Abcam)<br>Tyrosine Hydroxylase AB76442 Chicken 1:500 (Abcam)<br>Tyrosine Hydroxylase AB112 Rabbit (Abcam)
Validation	4-HNE (cat # HNE11-S) produced in rabbits.<br>Yoritaka A, 1996, PNAS 93(7):2696-2701, DOI: 10.1073/pnas.93.7.2696<br>McKim SE, 2002, Arch Biochem Biophy. 406, 40-46, DOI: 10.1016/s0003-9861(02)00425-3<br>Bido S, 2017, Scientific Rep. 7:7495 DOI: 10.1038/s41598-017-07181-0<br><br>ASC AL177 (cat. AG-25B-006) synthetic peptide corresponding to aa at the N terminal human Asc, source Rabbit.<br>Venegas C, 2017, Nature, 20;552(7685):355-361 DOI: 10.1038/nature25158<br><br>CD11b eFluor 450 M clone M1/70, (cat. 48-0112-82) The M1/70 monoclonal antibody reacts with mouse CD11b, the 165-170 kDa integrin alphaM<br>Zhang SR, 2018, JCI insight, 20;3(18):e121560 DOI: 10.1172/jci.insight.121560.<br><br>CD22 (cat. EPR20061) Rabbit monoclonal produced recombinantly (animal-free).<br>The validation is provided by the supplier (https://www.abcam.com/cd22-antibody-epr20061-ab207727.html)<br><br>CD3 (cat. MCA772GA) clone 1F4, mouse anti Rat, recognizes rat CD3 found on rat T-cells<br>Sanchez-Guajardo V, 2010, PLoS One 20;5(1):e8784 DOI: 0.1371/journal.pone.0008784.<br>Tanaka T, 1989, J Immunology, 15;142(8):2791-5 PMID: 2467940<br><br>CD45 PerCP-Cyanine5.5 clone 30-F11 (cat. 45-0451-82). The 30-F11 monoclonal antibody reacts with all isoforms of mouse CD45<br>MacPherson KP, 2017, Neurobiology of disease, 102:81-95 DOI: 10.1016/j.nbd.2017.02.010<br><br>CD68 (cat. MCA1967T) clone FA-11, rat anti mouse, recognizes mouse macrosialin, a heavily glycosylated transmembrane protein and murine homolog of human CD68.<br>Lazarini F, 2012, J Neuroscience, 32:3652-64, DOI: 10.1523/JNEUROSCI.6394-11.2012<br><br>GFAP (cat. GA524) Rabbit Anti-Glial Fibrillary Acidic Protein<br>Chucair-Elliott AJ, 2014, Glia, 62(9):1418-34 DOI: 10.1002/glia.22689<br><br>GFP (cat. ab13970) Chicken polyclonal to recombinant full length protein corresponding to GFP<br>Badimon A, 2020, Nature, 586:417-423, DOI: 10.1038/s41586-020-2777-8<br><br>GFP (cat. ab290) Rabbit polyclonal to full length protein corresponding to green fluorescent protein from Aequorea victoria.<br>Massaro G, 2020, Hum Mol Genet, 29:1933-1949, DOI: 10.1093/hmg/ddz317<br><br>Human alpha-synuclein (cat. 180215) clone LB509. Immunogenicity to Lewy Bodies purified from patients suffering dementia with Lewy Bodies. It reacts with human alpha-synuclein<br>Oueslati A, 2013, PNAS, 8;110(41):E3945-54, DOI: 10.1073/pnas.1309991110.<br><br>IBA1 (cat. 019-197441) rabbit polyclonal<br>Rauch JN, 2020, Nature, 580(7803):381-385 DOI: 10.1038/s41586-020-2156-5<br><br>IBA1 (cat. 234006) Polyclonal chicken purified antibody<br>Nortley R, 2019, Science, 20; 365(6450), DOI: 10.1126/science.aav9518<br><br>NeuN (cat. EPR12763) Rabbit monoclonal to NeuN - Neuronal Marker<br>Vrselja Z, 2019, Nature, 568:336-343 DOI: 10.1038/s41586-019-1099-1

	p62/SQSTM1 (cat. 48320) Rabbit polyclonal. Immunogenicity to genomic peptide made to c-t of the human p62/SQSTM1 protein (within residues 300-440). [Swiss-Prot Q13501].<br>Ciric D, 2018, Neuropathology, 38(5):468-474 DOI: 10.1111/neup.1250<br><br>pS129 alpha-synuclein (cat. EP1536Y) Rabbit monoclonal to alpha-synuclein (phospho S129). This antibody only detects alpha synuclein phosphorylated on Ser129<br>Schweighauser M, 2020, Nature, 585:464-469 DOI: 10.1038/s41586-020-2317-6<br><br>Tyrosine Hydroxylase (cat. AB76442) Chicken polyclonal to Tyrosine Hydroxylase<br>Frau R, 2019, Nat Neuroscience 22:1975-1985 DOI: 10.1038/s41593-019-0512-2<br><br>Tyrosine Hydroxylase (cat. AB112) Rabbit polyclonal to Tyrosine Hydroxylase<br>Lang Y, 2020, Aging, 12:7660-7678 DOI: 10.18632/aging.103028

Eukaryotic cell lines

Policy information about cell lines

Cell line source(s)	HEK293T: ATCC, Cat# CRL-3216.
Authentication	HEK293T were authenticated by the supplier
Mycoplasma contamination	The cell line was negative for mycoplama contamination (PCR-test)
Commonly misidentified lines (See ICLAC register)	No commonly misidentified cell lines were used.

Animals and other organisms

Policy information about studies involving animals; ARRIVE guidelines recommended for reporting animal research

Laboratory animals	9 weeks old males Slc6a3tm1(cre)Xz/J (DAT) were purchased from Jackson Laboratories;<br>9 weeks old males B6;FVB-Tg(Aldh1l1-cre/ERT2)1Khakh/J (CX3CR1) were purchased from Jackson Laboratories;<br>9 weeks old males wild type C57BL6 mice were purchased from Jackson Laboratories
Wild animals	This study does not involve wild animals
Field-collected samples	This study does not involve samples collected from the field
Ethics oversight	All procedures were performed according to protocols approved by the Ospedale San Raffaele internal institutional animal care and use committee (IACUC, Milan, Italy) and reported to the Italian Ministry of Health according to the European Commission Council Directive 2010/63/EU.

Note that full information on the approval of the study protocol must also be provided in the manuscript.

Human research participants

Policy information about studies involving human research participants

Population characteristics	Human brain autoptic tissue samples were obtained by the Multiple Sclerosis and Parkinson's Tissue Bank, Imperial College London, UK. No need of particular information since it is not a covariate-relevant population
Recruitment	The samples used in this study are post-mortem tissue. Healthy and diseased patients were selected based on their clinical symptoms and post-mortem confirmed by the presence of neuropathological markers.
Ethics oversight	For the purpose of this study, the use of post-mortem tissue did not need any ethical approval statement

Note that full information on the approval of the study protocol must also be provided in the manuscript.

Flow Cytometry

Plots

Confirm that:

- [x] The axis labels state the marker and fluorochrome used (e.g. CD4-FITC).
- [x] The axis scales are clearly visible. Include numbers along axes only for bottom left plot of group (a 'group' is an analysis of identical markers).
- [x] All plots are contour plots with outliers or pseudocolor plots.
- [x] A numerical value for number of cells or percentage (with statistics) is provided.

Methodology

Sample preparation	The brain nigral regions were carefully dissected from 2 subsequent 500µm-thick slices and the tissue directly subjected for the single cell dissociation. The dissociation was performed using Adult Brain Dissociation Kit and OctoMACS Separator (both from Miltenyi Biotec) following manufacturer's instructions. Cells were stained with 1:200 Zombie Aqua Fixable Viability Kit (Biolegend) and subsequently with anti-CD45 PerCP-Cy5.5 antibody and anti-CD11b eFluor450 antibody (all from eBioscience). Cells were sorted using a FACSAria Fusion (BD) and collected in PBS 0.04%
Instrument	FACS Aria Fusion (BD)
Software	FCS Express 6 Flow (De NovoSoftware)
Cell population abundance	The post-sorted cell population accounts for the 6-10% of the total. The purity of the samples has been confirmed by the single cell sequencing.
Gating strategy	FSC-A/FSC-H were used to exclude doublets and FSC-A/SSC-A were used to exclude debris. CD45+ and CD11b+ signals were used to select lymphocyte ad leukocyte population.

☒ Tick this box to confirm that a figure exemplifying the gating strategy is provided in the Supplementary Information.
